# Supplementary material for: MARTX toxin of Vibrio vulnificus induces RBC phosphatidylserine exposure that can contribute to thrombosis
Source: Nat Commun. 2022 Aug 17;13:4846. doi: 10.1038/s41467-022-32599-0 (PMC9385741; doi:10.1038/s41467-022-32599-0)
Supplement: Supplementary file 1 — Supplementary Information [file 41467_2022_32599_MOESM1_ESM.pdf]

# Supplementary information

## MARTX toxin of *Vibrio vulnificus* induces RBC phosphatidylserine exposure that can contribute to thrombosis

**Han Young Chung[1,2], Yiying Bian[3], Kyung-Min Lim[4], Byoung Sik Kim[5], and Sang Ho Choi[1,2*]**

[1] National Research Laboratory of Molecular Microbiology and Toxicology, Department of Agricultural Biotechnology, Seoul National University, Seoul 08826, Republic of Korea

[2] Center for Food and Bioconvergence, Seoul National University, Seoul 08826, Republic of Korea

[3] School of Public Health, China Medical University, Shenyang 110122, People's Republic of China

[4] College of Pharmacy, Ewha Womans University, Seoul 03760, Republic of Korea

[5] Department of Food Science and Engineering, Ewha Womans University, Seoul 03760, Republic of Korea

***Correspondence:** choish@snu.ac.kr

## Supplementary figure legends:

**Supplementary Fig. 1 Shape changes of RBCs infected with *V. vulnificus*.** RBCs were infected with *V. vulnificus* at various MOIs and incubated for 1 h. *V. vulnificus* only (left) and the shape changes of the human RBCs infected with *V. vulnificus* (red arrowheads) at various MOIs and incubated for 1 h (right) were analyzed by TEM. The representative images by TEM among 5 independent experiments were presented. Red arrowhead indicates *V. vulnificus*; white arrow, echinocyte; and white arrowhead, spherocyte. Scale bar is presented. MOI, multiplicity of infection; and TEM, transmission electron microscopy.

**Supplementary Fig. 2 Effects of *V. vulnificus* on the MV generation, and PS exposure of human RBCs. a, b** RBCs were infected with *V. vulnificus* at various MOIs and different incubation times. MV generation (**a**) and PS exposure (**b**) were determined (n=6, two-way analysis of variance (ANOVA) followed by Duncan's multiple range test). The means ± SE were calculated from at least five independent experiments. Control, uninfected; MOI, multiplicity of infection; PS, phosphatidylserine; and MV, microvesicle.

**Supplementary Fig. 3 Effect of purified Annexin V on thrombin generation in *V. vulnificus*-infected RBCs.** Human RBCs were incubated in the presence or absence of purified Annexin V for 10 min. And then, the pre-incubated RBCs were infected with *V. vulnificus* at 5, and 10 MOIs for 30 min, and thrombin generation were determined (n=5, two-tailed Student's *t*-test). The means ± SE. were calculated from at least five independent experiments. Control, uninfected; and MOI, multiplicity of infection.

**Supplementary Fig. 4 Characteristics of the *V. vulnificus* mutant strains used in the study. a** Schematic diagrams of the *rtxA* structural gene of the *V. vulnificus* WT, *rtxA*::*nptI*

mutant, and revertant strains. The *rtxA*::*nptI* mutant carries the *nptI* gene cassette inserted at the 5'-end of *rtxA* and produces no MARTX toxin. The revertant carries the WT *rtxA* gene restored from the *rtxA*::*nptI* by removing the *nptI* insertion as indicated with dimmed symbol, and thus produces the WT-MARTX toxin. Arrows indicate transcriptional directions of each gene. **b, c** Human RBCs were infected with different strains of *V. vulnificus* at an MOI of 10 and incubated for 1 h, and then PS exposure (**b**) (n=5, two-tailed Student's *t*-test) and thrombin generation (**c**) (n=6, two-tailed Student's *t*-test) were determined. **d** Human epithelial cells (HeLa cells, 1 x $10^5$ cells) originally purchased from ATCC (CCL-2) were infected with different strains of *V. vulnificus* at an MOI of 10 and incubated for 150 min, and then bleb formation (red arrowheads) was observed. Scale bar is presented. The means ± SE. were calculated from at least five independent experiments. WT, wild type; *ΔrtxA*, a mutant producing no MARTX toxin; *rtxA*::*nptI*, a mutant producing no MARTX toxin; Rev, a revertant producing the WT-MARTX toxin; *nptI*, aminoglycoside 3'-phosphotransferase gene; *EF-rtxA*, a mutant producing the EF-MARTX toxin; PS, phosphatidylserine; control, uninfected; and MOI, multiplicity of infection.

**Supplementary Fig. 1**

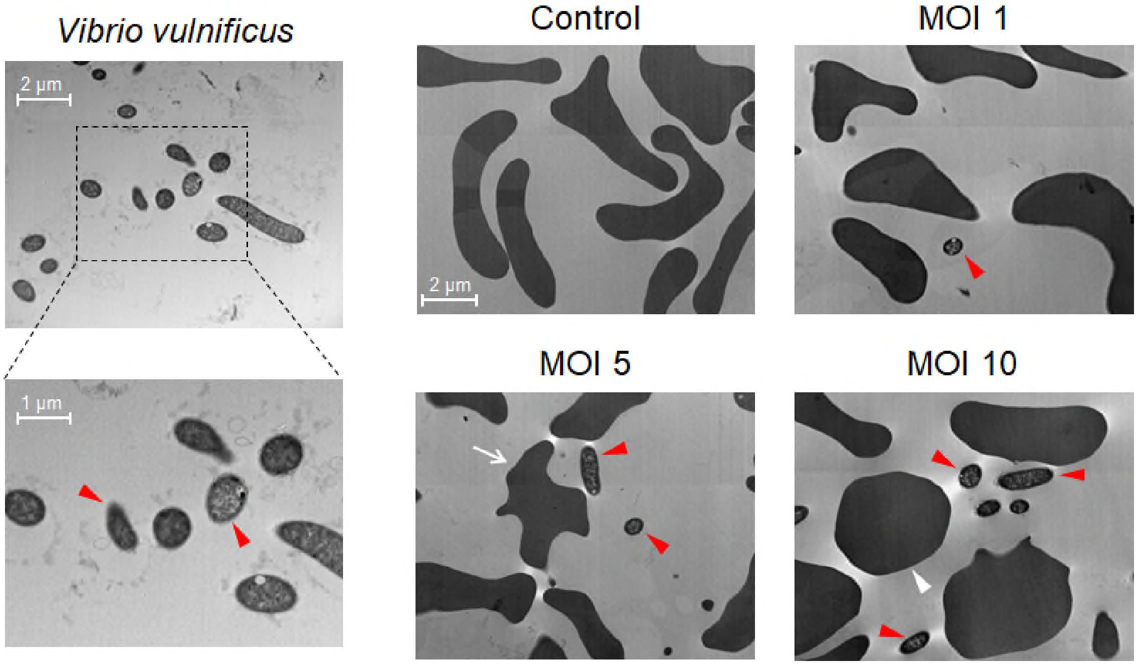

**Supplementary Fig. 2**

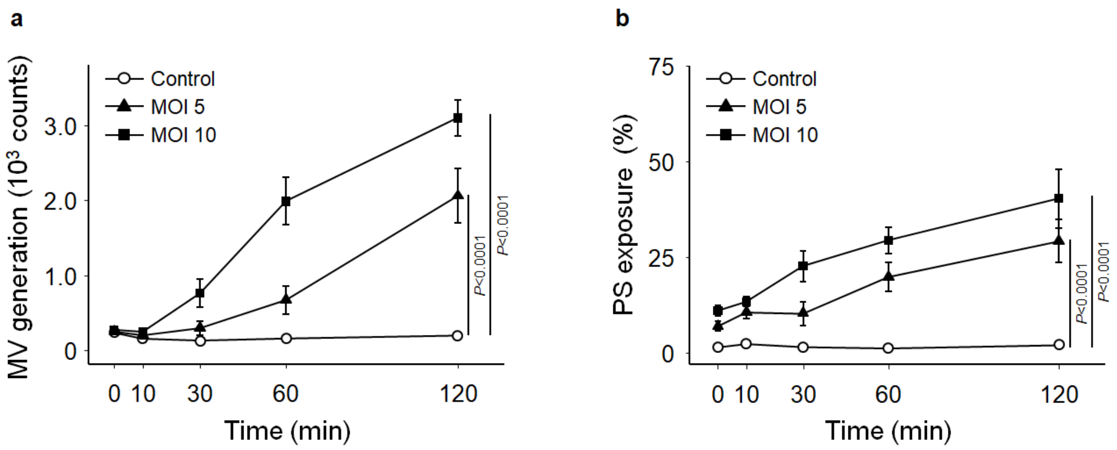

**Supplementary Fig. 3**

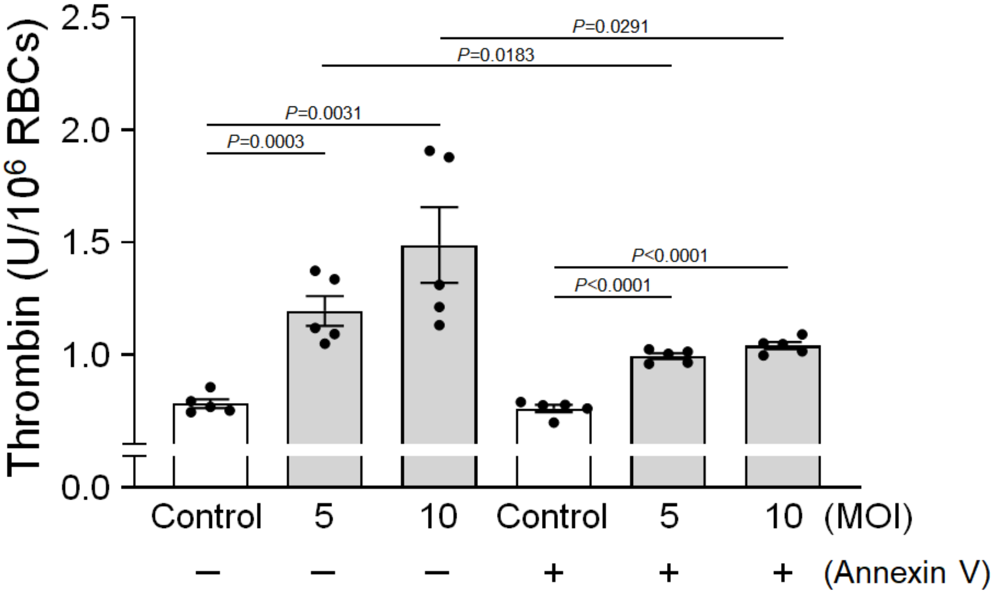

## Supplementary Fig. 4

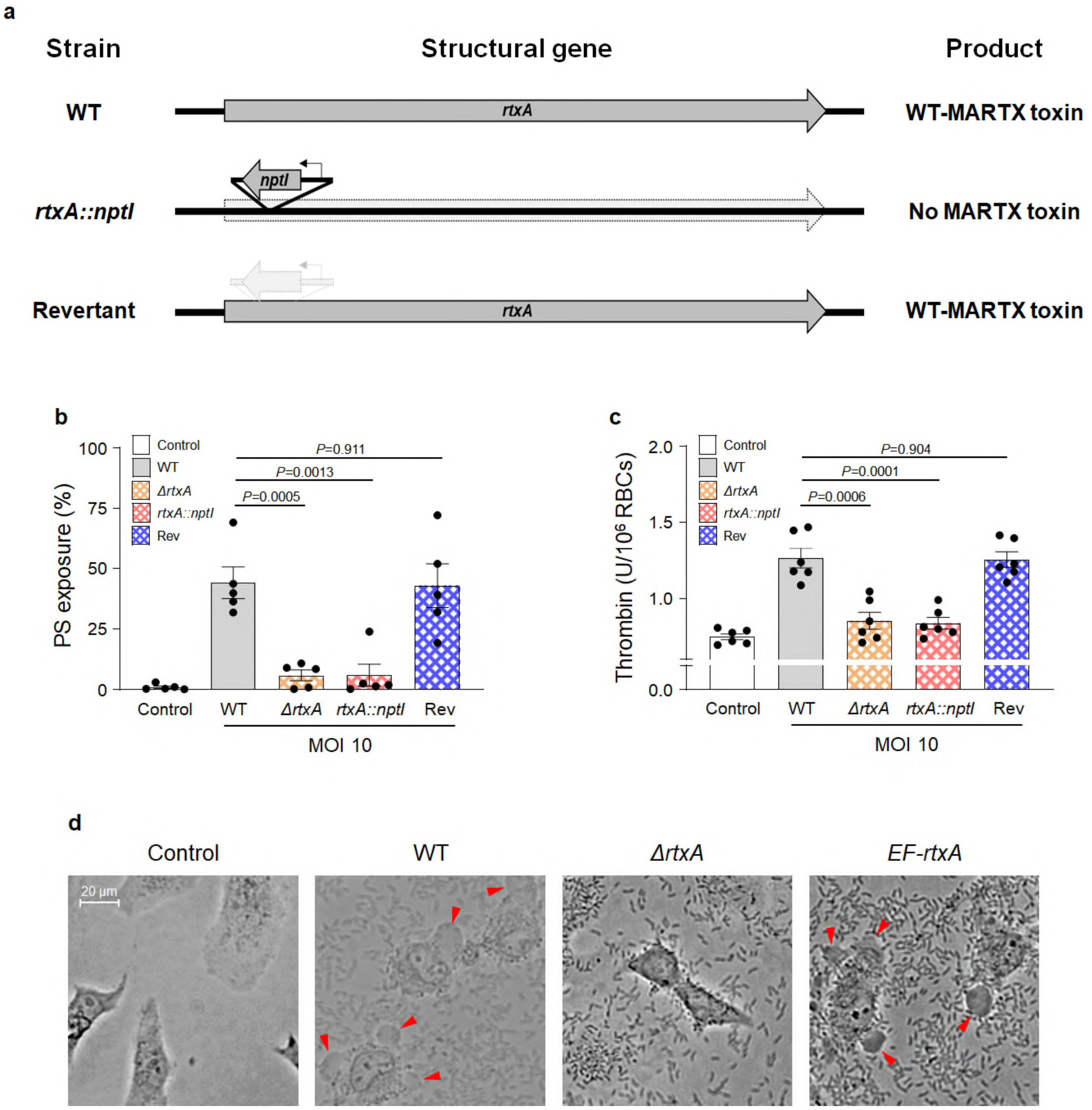

**Supplementary Table 1 Bacterial strains and plasmids used in this study**

| Strain or plasmid | Relevant characteristics[a] | Reference or source |
|---|---|---|
| **Bacterial strains** | | |
| *Vibrio vulnificus* | | |
| WT | Clinical isolate MO6-24/O, wild type, WT-MARTX toxin | (Wright et al., 1990)[1] |
| *ΔrtxA* | MO6-24/O with *ΔrtxA*::*nptI*, $Km^r$, no MARTX toxin | (Lee et al., 2007)[2] |
| *EF-rtxA* | MO6-24/O with *EF-rtxA*, EF-MARTX toxin | This study |
| *rtxA::nptI* | MO6-24/O with *rtxA*::*nptI*, $Km^r$, no MARTX toxin | (Lee et al., 2018)[3] |
| Revertant | MO6-24/O with the restored *rtxA*, $Km^s$, WT-MARTX toxin | (Lee et al., 2018)[3] |
| *Escherichia coli* | | |
| S17-1 λ pir | λ *pir* lysogen, *thi pro hsdR hsdM*$^+$ *recA* RP4-2 Tc::Mu-Km::Tn7, $Tp^R$, $Sm^R$, host for π-requiring plasmids, conjugal donor | (Simon et al., 1983)[4] |
| **Plasmid** | | |
| pDS132 | *oriR6K sacB oriT* RP4, $Cm^R$ | (Philippe et al., 2004)[5] |
| pDS_EF-MARTX | pDS132::*EF-rtxA*, $Cm^R$ | (Lee et al., 2019)[6] |

WT, wild type; [a] $Km^r$, kanamycin-resistant; EF, effector-free; $Km^s$, kanamycin-sensitive; $Tp^R$, Trimethoprim-resistant; $Sm^R$, streptomycin-resistant; $Cm^R$, chloramphenicol-resistant.

**Supplementary Table 2 List of differentially expressed genes in *V. vulnificus* exposed to human RBCs**

| Locus tag[a] | Annotation | RLE (DESeq2) | | $Log_2$ fold change | *P* value |
|---|---|---|---|---|---|
| | | Control | Exposed to RBCs | | |
| **Up-regulation[b]** | | | | | |
| **Amino acid transport and metabolism[c]** | | | | | |
| **VVMO6_01726** | Homoserine O-succinyltransferase | 136.78 | 369.50 | 1.43 | 2.56.E-28 |
| **VVMO6_03431** | 2-aminoethylphosphonate | 243.40 | 521.45 | 1.10 | 2.02E-22 |
| **VVMO6_03434** | ATP-binding protein | 311.33 | 781.19 | 1.33 | 6.42E-40 |
| **VVMO6_04200** | 2-keto-3-deoxy-D-arabino-Heptulosonate-7-phosphate synthase I alpha | 113.39 | 288.97 | 1.35 | 1.63E-20 |
| **VVMO6_04207** | Isochorismate pyruvate-lyase of siderophore biosynthesis | 32.15 | 94.69 | 1.56 | 2.88E-11 |
| **Carbohydrate transport and metabolism[c]** | | | | | |
| **VVMO6_02646** | Glycosyltransferase | 222.26 | 447.00 | 1.01 | 2.52E-16 |
| **VVMO6_03242** | Metabolite transporter | 935.99 | 2072.28 | 1.15 | 1.83E-41 |
| **Coenzyme transport and metabolism[c]** | | | | | |
| **VVMO6_01252** | Nicotinate phosphoribosyltransferase | 536.63 | 1319.58 | 1.30 | 3.94E-60 |
| **VVMO6_03382** | Radical SAM family protein HutW | 127.16 | 295.72 | 1.22 | 8.23E-18 |
| **Transcription[c]** | | | | | |
| **VVMO6_01093** | Transcriptional activator MetR | 70.83 | 252.16 | 1.83 | 4.33E-30 |
| **Replication, recombination and repair[c]** | | | | | |
| **VVMO6_03779** | RstA phage-related replication protein | 16.74 | 35.71 | 1.09 | 2.80E-03 |

| | | | | | |
|---|---|---|---|---|---|
| **Cell wall/membrane/envelope biogenesis**[c] | | | | | |
| **VVMO6_03380** | Ferric siderophore transport system periplasmic binding protein TonB | 7.31 | 17.36 | 1.25 | 1.00E-02 |
| **VVMO6_04039** | Putative outer membrane protein | 1853.30 | 4221.61 | 1.19 | 6.68E-38 |
| **Inorganic ion transport and metabolism**[c] | | | | | |
| **VVMO6_00202** | Manganese superoxide dismutase | 109.24 | 315.19 | 1.53 | 5.10E-25 |
| **VVMO6_04408** | Aerobactin siderophore receptor IutA | 99.93 | 209.56 | 1.07 | 3.22E-13 |
| **Secondary metabolites biosynthesis, transport and catabolism**[c] | | | | | |
| **VVMO6_04208** | 2,3-dihydroxybenzoate-AMP ligase | 64.23 | 214.36 | 1.74 | 2.16E-23 |
| **VVMO6_04209** | Aryl carrier domain | 5.54 | 15.06 | 1.44 | 1.74E-02 |
| **General function prediction only**[c] | | | | | |
| **VVMO6_01352** | Death on curing protein, Doc toxin | 2.16 | 7.04 | 1.71 | 4.35E-02 |
| **Intracellular trafficking, secretion, and vesicular transport**[c] | | | | | |
| **VVMO6_01206** | *flp* pilus assembly protein CpaB | 13.60 | 40.05 | 1.56 | 6.75E-06 |
| **Function unknown**[c] | | | | | |
| **VVMO6_01693** | Tricarboxylate transport protein TctC | 320.99 | 1479.75 | 2.20 | 4.36E-147 |
| **VVMO6_01696** | Tricarboxylate transport membrane protein TctA | 116.38 | 404.24 | 1.80 | 8.37E-46 |
| **Unclassified**[c] | | | | | |
| **VVMO6_01205** | Hypothetical protein | 15.46 | 31.96 | 1.05 | 3.10E-03 |
| **VVMO6_01356** | Hypothetical protein | 31.98 | 80.23 | 1.33 | 3.34E-07 |

| | | | | | |
|---|---|---|---|---|---|
| **VVMO6_01694** | Hypothetical protein | 3.98 | 40.23 | 3.34 | 6.40E-12 |
| **VVMO6_01695** | Tricarboxylate transport protein TctB | 32.15 | 142.11 | 2.14 | 2.95E-25 |
| **VVMO6_01834** | Predicted manganese transporter | 53.99 | 167.38 | 1.63 | 8.27E-19 |
| **VVMO6_02535** | Hypothetical protein | 23.50 | 48.17 | 1.04 | 3.65E-04 |
| **VVMO6_03008** | Hypothetical protein | 21.29 | 44.66 | 1.07 | 4.20E-04 |
| **VVMO6_03538** | Type II/IV secretion system ATPase TadZ/CpaE | 37.02 | 77.53 | 1.07 | 4.77E-06 |
| **VVMO6_03894** | Transposase | 4.80 | 13.46 | 1.49 | 2.18E-02 |
| **VVMO6_04161** | Hypothetical protein | 81.05 | 240.29 | 1.57 | 4.25E-22 |
| **Iron uptake**[d] | | | | | |
| **VVMO6_04199** | Siderophore biosynthesis | 120.07 | 278.71 | 1.21 | 2.39E-18 |
| **VVMO6_04201** | Catechol siderophore ABC transporter | 31.81 | 88.13 | 1.47 | 2.87E-09 |
| **VVMO6_04206** | Siderophore biosynthesis | 48.56 | 163.93 | 1.76 | 2.51E-21 |
| **VVMO6_04210** | Catechol siderophore ABC transporter | 98.78 | 216.15 | 1.13 | 1.56E-13 |
| **Toxin**[d] | | | | | |
| **VVMO6_03947** | MARTX toxin | 1121.50 | 2578.14 | 1.20 | 2.72E-43 |
| **Down-regulation**[b] | | | | | |
| **Energy production and conversion**[c] | | | | | |
| **VVMO6_01670** | Acylphosphate phosphohydrolase putative | 4.49 | 0.35 | -3.69 | 2.18E-02 |
| **VVMO6_02002** | Glutamate symport protein | 962.04 | 461.17 | -1.06 | 3.18E-36 |
| **VVMO6_02391** | Glutamate symport protein | 3187.71 | 967.56 | -1.72 | 2.21E-123 |
| **VVMO6_03181** | Oligopeptide transport ATP-binding protein OppF | 157.11 | 60.11 | -1.39 | 4.93E-13 |
| **VVMO6_03847** | Nitrite reductase | 109.18 | 46.40 | -1.23 | 8.32E-09 |
| **VVMO6_03866** | Nitrite reductase | 9012.01 | 4048.87 | -1.15 | 3.44E-05 |

| | | | | | |
|---|---|---|---|---|---|
| **VVMO6_04094** | Ferredoxin-type protein NapF | 310.68 | 119.27 | -1.38 | 1.11E-23 |
| **VVMO6_04096** | Periplasmic nitrate reductase precursor | 4850.36 | 1241.94 | -1.97 | 3.59E-161 |
| **VVMO6_04097** | Nitrate reductase cytochrome c550-type subunit | 263.20 | 60.19 | -2.13 | 7.57E-36 |
| **VVMO6_04098** | Cytochrome c-type protein NapC | 378.90 | 83.75 | -2.18 | 9.81E-50 |
| **VVMO6_04166** | L-lactate permease | 3883.93 | 1851.31 | -1.07 | 1.15E-57 |
| **Amino acid transport and metabolism[c]** | | | | | |
| **VVMO6_00054** | Ketol-acid reductoisomerase | 9904.53 | 4244.73 | -1.22 | 6.09E-75 |
| **VVMO6_00199** | Aspartate ammonia-lyase | 490.20 | 199.48 | -1.30 | 3.59E-31 |
| **VVMO6_01167** | Tyrosine-specific transport protein | 913.90 | 413.44 | -1.14 | 1.44E-36 |
| **VVMO6_01655** | Sodium/glycine symporter GlyP | 951.96 | 346.61 | -1.46 | 1.18E-53 |
| **VVMO6_02415** | Sodium/alanine symporter | 955.08 | 349.24 | -1.45 | 8.05E-53 |
| **VVMO6_02533** | Sodium/alanine symporter | 1281.58 | 456.23 | -1.49 | 2.88E-65 |
| **VVMO6_02972** | Amino acid ABC transporter | 7313.88 | 2351.55 | -1.64 | 2.70E-85 |
| **VVMO6_03045** | Sodium/dicarboxylate symporter | 1255.89 | 548.12 | -1.20 | 1.37E-48 |
| **VVMO6_03441** | Di-/tripeptide transporter | 80.95 | 26.87 | -1.59 | 1.89E-09 |
| **VVMO6_03488** | Serine transporter | 384.21 | 177.75 | -1.11 | 1.59E-18 |
| **Carbohydrate transport and metabolism[c]** | | | | | |
| **VVMO6_00328** | Ribulose-phosphate 3-epimerase | 359.94 | 173.56 | -1.05 | 1.76E-17 |
| **Coenzyme transport and metabolism[c]** | | | | | |
| **VVMO6_00730** | Multidrug efflux pump component MtrF | 160.67 | 65.49 | -1.29 | 8.42E-13 |
| **VVMO6_02721** | Uroporphyrinogen-III methyltransferase | 6674.09 | 3319.38 | -1.01 | 2.96E-33 |

| | | | | | |
|---|---|---|---|---|---|
| **Lipid transport and metabolism[c]** | | | | | |
| **VVMO6_00780** | Butyryl-CoA dehydrogenase | 2445.81 | 1085.24 | -1.17 | 9.99E-38 |
| **Translation, ribosomal structure and biogenesis[c]** | | | | | |
| **VVMO6_02205** | Endoribonuclease L-PSP | 39.83 | 17.42 | -1.19 | 4.79E-4 |
| **Transcription[c]** | | | | | |
| **VVMO6_03062** | Transcriptional regulator | 121.32 | 57.39 | -1.08 | 9.48E-08 |
| **VVMO6_04439** | Transcriptional regulator | 490.02 | 235.65 | -1.06 | 3.40E-21 |
| **Cell wall/membrane/envelope biogenesis[c]** | | | | | |
| **VVMO6_03345** | Capsular polysaccharide synthesis enzyme CpsI | 104.12 | 51.71 | -1.01 | 2.34E-06 |
| **VVMO6_04183** | Lipoprotein | 436.29 | 216.01 | -1.01 | 8.75E-19 |
| **Posttranslational modification, protein turnover, chaperones[c]** | | | | | |
| **VVMO6_00209** | Heat shock protein 60 family co-chaperone Gro | 396.09 | 101.56 | -1.96 | 7.93E-44 |
| **VVMO6_00210** | Heat shock protein 60 family chaperone GroEL | 9753.06 | 3465.84 | -1.49 | 1.49E-74 |
| **VVMO6_00297** | ATP-dependent hsl protease ATP-binding subunit HslU | 2433.27 | 1057.85 | -1.20 | 2.10E-53 |
| **VVMO6_00298** | ATP-dependent protease HslV | 553.43 | 150.15 | -1.88 | 9.16E-48 |
| **VVMO6_02385** | Chaperone protein DnaK | 10047.69 | 4211.13 | -1.25 | 4.91E-65 |
| **VVMO6_02477** | ClpB protein | 6924.19 | 3211.64 | -1.11 | 3.10E-59 |
| **VVMO6_02966** | 16 kDa heat shock protein A | 2949.54 | 632.75 | -2.22 | 8.41E-33 |
| **VVMO6_04438** | Glutaredoxin 3 | 9.24 | 2.54 | -1.86 | 2.41E-02 |
| **VVMO6_04522** | Heat shock protein 60 family chaperone GroEL | 7113.21 | 2770.01 | -1.36 | 1.23E-51 |

| | | | | | |
|---|---|---|---|---|---|
| **VVMO6_04523** | Heat shock protein 60 family co-chaperone GroES | 344.60 | 90.14 | -1.93 | 1.29E-32 |
| **Inorganic ion transport and metabolism[c]** | | | | | |
| **VVMO6_00653** | Chromate transport protein ChrA | 102.39 | 50.56 | -1.02 | 1.47E-06 |
| **VVMO6_00937** | Lead, cadmium, zinc and mercury transporting ATPase | 1652.00 | 799.21 | -1.05 | 2.12E-28 |
| **VVMO6_02186** | Ferrous iron transport protein A | 10.23 | 2.29 | -2.16 | 8.89.E-03 |
| **VVMO6_02280** | Lead, cadmium, zinc and mercury transporting ATPase | 839.91 | 407.86 | -1.04 | 4.27E-22 |
| **VVMO6_02509** | Sodium-dependent phosphate transporter | 6498.65 | 2385.24 | -1.45 | 2.25E-86 |
| **VVMO6_03867** | Nitrate ABC transporter ATP-binding protein | 2859.99 | 995.43 | -1.52 | 8.29E-09 |
| **VVMO6_03868** | Nitrate ABC transporter permease protein | 2815.34 | 668.68 | -2.07 | 6.61E-38 |
| **VVMO6_03869** | Nitrate ABC transporter nitrate-binding protein | 4960.46 | 1164.12 | -2.09 | 2.22E-27 |
| **VVMO6_04095** | Periplasmic nitrate reductase component NapD | 442.22 | 123.19 | -1.84 | 6.69E-32 |
| **Secondary metabolites biosynthesis, transport and catabolism[c]** | | | | | |
| **VVMO6_01992** | Hypothetical protein | 411.85 | 174.84 | -1.24 | 5.60E-25 |
| **General function prediction only[c]** | | | | | |
| **VVMO6_00722** | Sodium-dependent transporter | 878.74 | 335.98 | -1.39 | 5.36E-48 |
| **VVMO6_02367** | Xanthine/uracil/thiamine/ascorbate permease family protein | 620.68 | 288.94 | -1.10 | 1.17E-26 |
| **VVMO6_04325** | Phospholipid-binding protein | 282.59 | 39.67 | -2.83 | 1.73E-48 |
| **VVMO6_04520** | Multimeric flavodoxin WrbA | 15.05 | 6.39 | -1.24 | 2.32E-02 |
| **VVMO6_04524** | Acetyltransferase | 43.92 | 20.73 | -1.08 | 1.02E-03 |
| **Signal transduction mechanisms[c]** | | | | | |

| | | | | | |
|---|---|---|---|---|---|
| **VVMO6_03870** | Putative two-component response regulatory protein | 579.57 | 129.13 | -2.17 | 2.77E-73 |
| **Defense mechanisms**[c] | | | | | |
| **VVMO6_00539** | ABC-type multidrug transport system | 227.40 | 78.06 | -1.54 | 1.19E-21 |
| **VVMO6_03667** | Multidrug resistance protein A | 178.81 | 72.97 | -1.29 | 1.47E-12 |
| **VVMO6_04387** | Membrane fusion component of tripartite multidrug resistance system | 15.39 | 7.15 | -1.11 | 3.76E-02 |
| **Function unknown**[c] | | | | | |
| **VVMO6_00408** | Predicted transmembrane protein | 40.21 | 18.84 | -1.09 | 8.44E-04 |
| **VVMO6_00453** | Hypothetical protein | 559.71 | 247.17 | -1.18 | 5.74E-27 |
| **VVMO6_00887** | Hypothetical protein | 108.91 | 50.59 | -1.11 | 1.3E-07 |
| **VVMO6_01713** | Hypothetical protein | 9.35 | 2.78 | -1.75 | 3.05E-02 |
| **VVMO6_02105** | Membrane protein | 125.60 | 60.84 | -1.05 | 2.50E-07 |
| **VVMO6_03810** | Hypothetical protein | 39.17 | 18.91 | -1.05 | 1.85E-03 |
| **VVMO6_04285** | Membrane protein | 14.34 | 5.21 | -1.46 | 1.24E-02 |
| **VVMO6_04437** | Membrane protein | 21.42 | 10.31 | -1.05 | 1.64E-02 |
| **Unclassified**[c] | | | | | |
| **VVMO6_00939** | Hypothetical protein | 824.70 | 317.42 | -1.38 | 2.46E-45 |
| **VVMO6_01196** | Hypothetical protein | 167.49 | 58.27 | -1.52 | 3.90E-14 |
| **VVMO6_01874** | Hypothetical protein | 236.18 | 99.02 | -1.25 | 2.31E-12 |
| **VVMO6_01899** | Hypothetical protein | 18.43 | 8.85 | -1.06 | 4.03E-02 |
| **VVMO6_02992** | Permease of the major facilitator superfamily | 19.07 | 9.16 | -1.06 | 3.28E-02 |
| **VVMO6_03146** | Hypothetical protein in aerobactin uptake cluster | 25.32 | 11.93 | -1.09 | 9.13E-03 |
| **VVMO6_03548** | Pyruvate/2-oxoglutarate dehydrogenase | 15.93 | 7.18 | -1.15 | 3.15E-02 |

| | | | | | |
|---|---|---|---|---|---|
| | complex dihydrolipoamide dehydrogenase component | | | | |
| **VVMO6_03986** | Hypothetical protein | 16.61 | 7.29 | -1.19 | 3.83E-02 |
| **VVMO6_04154** | Permease of the major facilitator superfamily | 40.51 | 19.02 | -1.09 | 1.48E-03 |
| **VVMO6_04217** | Hypothetical protein | 10.42 | 3.36 | -1.63 | 2.70E-02 |
| **VVMO6_04375** | Acyl-CoA synthetase | 175.34 | 82.71 | -1.08 | 1.09E-10 |
| **VVMO6_04432** | Hypothetical protein | 20.18 | 9.30 | -1.12 | 2.04E-02 |
| **VVMO6_04506** | Hypothetical protein | 9.45 | 2.88 | -1.71 | 2.55E-02 |
| **VVMO6_04521** | Hypothetical protein | 28.86 | 9.86 | -1.55 | 5.05.E-04 |
| **Antiphagocytosis[d]** | | | | | |
| **VVMO6_03346** | Capsular polysaccharide | 44.61 | 16.59 | -1.43 | 2.11E-05 |
| **VVMO6_03350** | Capsular polysaccharide | 149.86 | 52.07 | -1.53 | 1.36E-14 |
| **VVMO6_03351** | Capsular polysaccharide | 595.28 | 230.69 | -1.37 | 1.17E-26 |
| **Chemotaxis and motility[d]** | | | | | |
| **VVMO6_02252** | Flagella | 3175.42 | 1519.97 | -1.06 | 4.24E-54 |

[a] Locus tags are based on the database for the *V. vulnificus* MO6-24/O genome (GenBank accession numbers: CP002469 and CP002470, https://www.ncbi.nlm.nih.gov/).

[b] $|\mathrm{Log}_2$ fold change$| \geq 1.0$, and $P$ value $\leq 0.05$.

[c] Clusters of orthologous genes (COG, http://weizhong-lab.ucsd.edu/webMGA/) category[7].

[d] Virulence Factor Database (VFDB, http://www.mgc.ac.cn/VFs/) category[8].

# Supplementary References

1. Wright, A. C., Simpson, L. M., Oliver, J. D. & Morris, J. G. Phenotypic evaluation of acapsular transposon mutants of *Vibrio vulnificus*. *Infect. Immun*. **58**, 1769–1773 (1990).
2. Lee, J. H. *et al*. Identification and characterization of the *Vibrio vulnificus rtxA* essential for cytotoxicity *in vitro* and virulence in mice. *J. Microbiol*. **45**, 146–52 (2007).
3. Lee, A. *et al. Vibrio vulnificus* RtxA is a major factor driving inflammatory T helper type 17 cell responses *in vitro* and *in vivo*. *Front. Immunol.* **9**, 1–11 (2018).
4. Simon, R., Priefer, U., & Puhler, A. A broad host range mobilization system for *in vivo* genetic engineering: transposon mutagenesis in Gram-negative bacteria. *BioTechnology* **1**, 784–7912 (1983).
5. Philippe, N., Alcaraz, J.-P., Coursange, E., Geiselmann, J. & Schneider, D. Improvement of pCVD442, a suicide plasmid for gene allele exchange in bacteria. *Plasmid* **51**, 246–255 (2004).
6. Lee, Y. *et al.* Makes caterpillars floppy-like effector-containing MARTX toxins require host ADP-ribosylation factor (ARF) proteins for systemic pathogenicity. *Proc. Natl. Acad. Sci. U. S. A.* **116**, 18031–18040 (2019).
7. Wu, S., Zhu, Z., Fu, L., Niu, B. & Li, W. WebMGA: A customizable web server for fast metagenomic sequence analysis. *BMC Genomics* **12**, (2011).
8. Liu, B., Zheng, D., Jin, Q., Chen, L. & Yang, J. VFDB 2019: A comparative pathogenomic platform with an interactive web interface. *Nucleic Acids Res.* **47**, D687–D692 (2019).
