# Supplementary material for: MARTX toxin of Vibrio vulnificus induces RBC phosphatidylserine exposure that can contribute to thrombosis
Source: Nat Commun. 2022 Aug 17;13:4846. doi: 10.1038/s41467-022-32599-0 (PMC9385741; doi:10.1038/s41467-022-32599-0)
Supplement: Supplementary file 3 — Reporting Summary [file 41467_2022_32599_MOESM3_ESM.pdf]

nature portfolio

Corresponding author(s): S. H. Choi

Last updated by author(s): Aug 2, 2022

Reporting Summary

Nature Portfolio wishes to improve the reproducibility of the work that we publish. This form provides structure for consistency and transparency in reporting. For further information on Nature Portfolio policies, see our Editorial Policies and the Editorial Policy Checklist.

Statistics

For all statistical analyses, confirm that the following items are present in the figure legend, table legend, main text, or Methods section.

n/a	Confirmed	
☐	☒	The exact sample size (*n*) for each experimental group/condition, given as a discrete number and unit of measurement
☐	☒	A statement on whether measurements were taken from distinct samples or whether the same sample was measured repeatedly
☐	☒	The statistical test(s) used AND whether they are one- or two-sided<br>*Only common tests should be described solely by name; describe more complex techniques in the Methods section.*
☒	☐	A description of all covariates tested
☒	☐	A description of any assumptions or corrections, such as tests of normality and adjustment for multiple comparisons
☐	☒	A full description of the statistical parameters including central tendency (e.g. means) or other basic estimates (e.g. regression coefficient) AND variation (e.g. standard deviation) or associated estimates of uncertainty (e.g. confidence intervals)
☐	☒	For null hypothesis testing, the test statistic (e.g. *F*, *t*, *r*) with confidence intervals, effect sizes, degrees of freedom and *P* value noted<br>*Give P values as exact values whenever suitable.*
☒	☐	For Bayesian analysis, information on the choice of priors and Markov chain Monte Carlo settings
☒	☐	For hierarchical and complex designs, identification of the appropriate level for tests and full reporting of outcomes
☒	☐	Estimates of effect sizes (e.g. Cohen's *d*, Pearson's *r*), indicating how they were calculated

Our web collection on statistics for biologists contains articles on many of the points above.

Software and code

Policy information about availability of computer code

Data collection	Data were collected as described in the manuscript.<br>Tecan Spark (Tecan) was used to measure the detection of hemolytic activity and prothrombinase assay.<br>Scanning electron microscopy (SEM) (Merlin Compact FE-SEM, Zeiss) was used to acquire the SEM images.<br>Confocal microscopy equipped with an argon laser (TCS SP8, Leica) was used to acquire the confocal images.<br>Transmission electron microscopy (TEM) (JEM-1010, JEOL) was used to acquire the TEM images.<br>FACSCalibur flow cytometer (Becton Dickinson) was used to acquire flow cytometry data using Cell Quest Pro software v.6.0.<br>Fluorescence Microscopy (Carl Zeiss, Axiovert 200M) was used to acquire the adherence and aggregation of RBCs to EC and self-aggregation images.<br>Transcriptome data was collected using the Illumina platform sequencing on a NovaSeq6000 instrument.<br>Stemi 305 stereomicroscope (Zeiss) equipped with an Axiocam 105 color camera (Zeiss) was used to acquire thrombus images.
Data analysis	For transcriptome analysis, the RLE values were normalized and analyzed statistically using DESeq2 v.1.26.0 to identify differentially expressed genes ($P < 0.05$).<br>Flow cytometry data were analyzed in FACSCalibur flow cytometer.<br>Statistical analysis was performed using SigmaPlot v.12.

For manuscripts utilizing custom algorithms or software that are central to the research but not yet described in published literature, software must be made available to editors and reviewers. We strongly encourage code deposition in a community repository (e.g. GitHub). See the Nature Portfolio guidelines for submitting code & software for further information.

Data

Policy information about availability of data

All manuscripts must include a data availability statement. This statement should provide the following information, where applicable:

- Accession codes, unique identifiers, or web links for publicly available datasets
- A description of any restrictions on data availability
- For clinical datasets or third party data, please ensure that the statement adheres to our policy

Total mRNA sequences from transcriptome analysis have been deposited in the NCBI Sequence Read Archive (SRA; http://www.ncbi.nlm.nih.gov/sra) under accession numbers SRR14307536, SRR14307537, SRR14307538, SRR14307539, SRR14307540, and SRR14307541.

Link for SRA data

SRR14307536 : https://trace.ncbi.nlm.nih.gov/Traces/study/?acc=SRR14307536%20&o=acc_s%3Aa

SRR14307537 : https://trace.ncbi.nlm.nih.gov/Traces/study/?acc=SRR14307537%20&o=acc_s%3Aa

SRR14307538 : https://trace.ncbi.nlm.nih.gov/Traces/study/?acc=SRR14307538%20&o=acc_s%3Aa

SRR14307539 : https://trace.ncbi.nlm.nih.gov/Traces/study/?acc=SRR14307539%20&o=acc_s%3Aa

SRR14307540 : https://trace.ncbi.nlm.nih.gov/Traces/study/?acc=SRR14307540&o=acc_s%3Aa

SRR14307541 : https://trace.ncbi.nlm.nih.gov/Traces/study/?acc=SRR14307541&o=acc_s%3Aa

Human research participants

Policy information about studies involving human research participants and Sex and Gender in Research.

Reporting on sex and gender	Healthy male volunteers were recruited for 5 mL blood donation. Male participants were identified via database available at Health Service Center, Seoul National University and recruited by contact with the study investigators. The purpose of our study was briefly explained to participants. All participants provided written informed consent before enrollment for the study.
Population characteristics	Healthy male volunteers which were 20-30 years old, reflect the Korean population in south part of Seoul Korea.
Recruitment	Participants were recruited on a voluntary basis via database available at Health Service Center of Seoul National University. When recruiting, the purpose of our study were explained to the participants. It is not likely to have any bias on the study results. Compensation for the blood donated was about US$ 25 for 5 mL blood.
Ethics oversight	With the approval from the Ethics Committee of Health Service Center at Seoul National University (IRB No. 1909/001-007), all participants provided written informed consent before enrollment for the study.

Note that full information on the approval of the study protocol must also be provided in the manuscript.

Field-specific reporting

Please select the one below that is the best fit for your research. If you are not sure, read the appropriate sections before making your selection.

☒ Life sciences ☐ Behavioural & social sciences ☐ Ecological, evolutionary & environmental sciences

For a reference copy of the document with all sections, see nature.com/documents/nr-reporting-summary-flat.pdf

Life sciences study design

All studies must disclose on these points even when the disclosure is negative.

Sample size	Statistical method were not used to pre-determine the sample size. Sample sizes of most in vitro experiments are 5 and more based on previous studies (1,2).<br>For in vivo study, the number of rats in each group (n=5-7) were used for statistical analysis, which is sufficient to give a significance and reproducibility.<br>RNA-seq analysis was performed with three biological replicates, which we considered to be sufficient due to low variability.<br><br>1. Bian Y. et al. Dapsone Hydroxylamine, an Active Metabolite of Dapsone, Can Promote the Procoagulant Activity of Red Blood Cells and Thrombosis. Toxicol. Sci. 172(2):435-444, 2019.

2. Noh JY. et al. Procoagulant and prothrombotic activation of human erythrocytes by phosphatidic acid. Am. J. Physiol. Heart Circ. Physiol. 299(2):H347-55, 2010.

Data exclusions	No data were excluded form the analyses.
Replication	All the data were obtained based upon at least five or more biologically independent human or animal samples and successfully replicated. RNA-seq analysis was performed only three independent experiments.
Randomization	For in vivo experiments, rats in the similar weight range were randomly assigned into different groups.<br>For in vitro experiments, RBC samples isolated from healthy human volunteer were randomly allocated into different groups.
Blinding	Investigators were blinded for in vivo experiments, since two or three animals in each group were underwent surgery without knowing the treatment group. After completion of animal experiments, all the data were collected for statistically analysis.<br>For RNA-seq analysis, the investigators were blinded for the treatment.<br>For other in vitro experiments, blinding was not performed. The quantitative measurements for optical density, fluorescence intensity, flow cytometry, etc. do not require investigator-based evaluation. We considered that blinding for in vitro experiment was not necessary.

Reporting for specific materials, systems and methods

We require information from authors about some types of materials, experimental systems and methods used in many studies. Here, indicate whether each material, system or method listed is relevant to your study. If you are not sure if a list item applies to your research, read the appropriate section before selecting a response.

Materials & experimental systems

n/a	Involved in the study
☐	☒ Antibodies
☐	☒ Eukaryotic cell lines
☒	☐ Palaeontology and archaeology
☐	☒ Animals and other organisms
☒	☐ Clinical data
☒	☐ Dual use research of concern

Methods

n/a	Involved in the study
☒	☐ ChIP-seq
☐	☒ Flow cytometry
☒	☐ MRI-based neuroimaging

Antibodies

Antibodies used	PE Mouse Anti-Human CD235a (Anti-glycophorin-A-PE) (BD Biosciences, 555570, 1:20 dilution), and Annexin V-FITC (BD Biosciences, 5564191,1:20 dilution) were used in the flow cytometry analysis. For the microscopic analysis, PE Mouse Anti-Human CD235a (Anti-glycophorin-A-PE) (BD Biosciences, 555570, 1:100 dilution), and Annexin V-FITC (BD Biosciences, 5564191,1:50 dilution) were used.
Validation	Antibodies were all validated by the manufacturer.<br>Further information can be obtained on the vendor website with the following links:<br><br>PE Mouse Anti-Human CD235a (Anti-glycophorin-A-PE) (BD Biosciences, 555570)<br>https://www.bdbiosciences.com/ko-kr/products/reagents/flow-cytometry-reagents/research-reagents/single-color-antibodies-ruo/pe-mouse-anti-human-cd235a.555570<br><br>Annexin V-FITC (BD Biosciences, 556419)<br>https://www.bdbiosciences.com/en-au/products/reagents/flow-cytometry-reagents/research-reagents/single-color-antibodies-ruo/fitc-annexin-v.556419

Eukaryotic cell lines

Policy information about cell lines and Sex and Gender in Research

Cell line source(s)	Human umbilical vein endothelial cells (HUVEC, Lonza, C2517A)<br>HeLa Cells, ATCC, CCL-2
Authentication	Since HUVEC and HeLa cells were originally purchased from Lonza and ATCC, respectively, the cell lines were not authenticated prior to use. The morphological feature of the cell lines appeared as expected.
Mycoplasma contamination	Cells were negative for mycoplasma contamination.
Commonly misidentified lines<br>(See ICLAC register)	No commonly misidentified cell lines were used.

Animals and other research organisms

Policy information about studies involving animals; ARRIVE guidelines recommended for reporting animal research, and Sex and Gender in Research

Laboratory animals	Eight weeks-old male SD rats were housed and kept on a regular 12-h light/dark cycle, room temperature: 20-25?, relative humidity: 50-70%. Food and water were available ad libitum.
Wild animals	The study did not involve any wild animals.
Reporting on sex	Only male SD rats were used in order to minimize the effect of hormonal fluctuations in this investigation.
Field-collected samples	The study did not involve any samples collected from the field.
Ethics oversight	All the protocols used for animal experiments were approved by the Ethics Committee of Animal Service Center at Seoul National University (SNU-170417-27-5).

Note that full information on the approval of the study protocol must also be provided in the manuscript.

Flow Cytometry

Plots

Confirm that:

☒ The axis labels state the marker and fluorochrome used (e.g. CD4-FITC).

☒ The axis scales are clearly visible. Include numbers along axes only for bottom left plot of group (a 'group' is an analysis of identical markers).

☒ All plots are contour plots with outliers or pseudocolor plots.

☒ A numerical value for number of cells or percentage (with statistics) is provided.

Methodology

Sample preparation	Human blood was collected from healthy male donors using a vacutatiner with acid citrate dextrose (ACD) on the day of each experiment. Buffy coat were removed by aspiration after centrifugation at 200 g for 15 min. Packed human RBCs were washed 2 times with phosphate buffered saline and once more with Ringer's solution. The washed RBCs were resuspended in Ringer's solution to a concentration of 1 x 10^8 cells/ml, in the presencce of 1mM CaCl2. After RBCs were infected with V. vulnificus, RBCs in incubation medium were stained directly for flow cytometry analysis.
Instrument	FACSCalibur flow cytometer (Bacton Dickinson)
Software	Cell Quest Pro software v.6.0 (Becton Dickinson) was used to acquire flow cytometry data.
Cell population abundance	All the cells were red blood cells. We examined the changing of the anti-glycophorin-A-PE (as RBC identifier) expression.
Gating strategy	With isolated RBCs from whole blood, FSC and SSC were used to test relative red cell size and cell granularity. RBCs (6-8 um) and MV (< 1 um) could be seperated by the relative size of cells (FSC) and graunularity (SSC). Annexin V-FITC was used to identify the PS exposure while anti-glycophorin A-PE was used to identify the RBCs. The gating is shown in Figure 2a. No other gating was performed. Fluorescence intensity was collected and the mean fluorescence intensity of each read is depicted in the graphs.

☒ Tick this box to confirm that a figure exemplifying the gating strategy is provided in the Supplementary Information.
